# A PEGylated Chitosan as Gel Polymer Electrolyte for Lithium Ion Batteries

**DOI:** 10.3390/polym14214552

**Published:** 2022-10-27

**Authors:** Anqi Wang, Yue Tu, Sijie Wang, Hongbing Zhang, Feng Yu, Yong Chen, De Li

**Affiliations:** 1 State Key Laboratory of Marine Resource Utilization in South China Sea, Hainan Provincial Key Laboratory of Research on Utilization of Si-Zr-Ti Resources, Hainan University, Haikou 570228, China; 2 Guangdong Key Laboratory for Hydrogen Energy Technologies, School of Materials Science and Hydrogen Energy, Foshan University, Foshan 528000, China

**Keywords:** PEGylated chitosan, gel polymer electrolyte, lithium ion batteries

## Abstract

Due to their safety and sustainability, polysaccharides such as cellulose and chitosan have great potential to be the matrix of gel polymer electrolytes (GPE) for lithium-based batteries. However, they easily form hydrogels due to the large numbers of hydrophilic hydroxyl or amino functional groups within their macromolecules. Therefore, a polysaccharide-based amphiphilic gel, or organogel, is urgently necessary to satisfy the anhydrous requirement of lithium ion batteries. In this study, a PEGylated chitosan was initially designed using a chemical grafting method to make an GPE for lithium ion batteries. The significantly improved affinity of PEGylated chitosan to organic liquid electrolyte makes chitosan as a GPE for lithium ion batteries possible. A reasonable ionic conductivity (1.12 × 10^−3^ S cm^−1^) and high lithium ion transport number (0.816) at room temperature were obtained by replacing commercial battery separator with PEG-grafted chitosan gel film. The assembled Li/GPE/LiFePO_4_ coin cell also displayed a high initial discharge capacity of 150.8 mA h g^−1^. The PEGylated chitosan-based GPE exhibits great potential in the field of energy storage.

## 1. Introduction

Lithium ion batteries (LIBs) are considered to be the most promising energy storage device due to their high energy density, long cycle life and environmental friendliness; they have been widely used in our daily lives [1,2,3]. Carbonate ester-based liquid electrolytes are responsible for the high ionic conductivity and good interfacial compatibility of LIBs [4,5]. However, LIBs are subject to serious safety issues such as leakage of electrolyte, thermal runaway and explosion, which can be attributed to the use of highly volatile and flammable liquid electrolytes, and the resulting growth of lithium dendrites [5,6,7]. In order to solve the safety problems, much research in recent years has been focused on solid polymer electrolytes (SPEs) because of their solid state and nonflammability [7,8,9,10]. However, SPEs also suffer from some problems, such as high interfacial resistance and low ionic conductivity [11]. In contrast, gel polymer electrolytes (GPEs) [12,13], which combine the advantages of both SPEs and liquid electrolytes, are considered a practical alternative for improving the safety of LIBs [14].

In GPEs, the high-molecular-weight polymers act as skeletons [14,15], ensuring good mechanical strength, high thermal stability and excellent electrochemical stability [16]; meanwhile, the liquid electrolytes can plasticize polymers, which endow GPEs with high ionic conductivity [17]. Over the past few decades, several typical gel polymer matrixes such as polyethylene oxide (PEO) [18,19], polyacrylonitrile (PAN) and poly (methyl methacrylate) (PMMA) [20,21,22,23,24], have been extensively investigated; among these, PEO and its derivatives have received a lot of attention due to the presence of ionic transporting groups in them, i.e., ether linkages (−C−O−C−) in their molecules [25]. However, on the one hand, conventional GPEs often have relatively lower Li^+^ transference numbers at room temperature; on the other hand, the production and use of these synthetic polymers often has a negative impact on the environment [26], which hinders further commercial application of GPEs [27]. Therefore, further research is necessary in order to obtain an environmentally friendly GPE with excellent lithium ion transport properties through simple and low-cost methods. Most significantly, GPEs that are synthesized from eco-friendly raw materials and green processes will be helpful to the remission of environmental problems [28,29,30].

For the above reasons, some researchers have turned their attention to natural polymeric materials in recent years [31]. Natural biomass polysaccharides such as cellulose [32,33], chitosan and starch have been reported as matrixes of polymer electrolytes [34]. Among them, chitosan (CS) is a linear polysaccharide that is derived from chitin in crustaceans and other animals and plants (e.g., crustacean cells, insect exoskeletons and fungal cell walls) [35]. Chitosan is well known to be the second most abundant and renewable biopolymer after cellulose. Heretofore, various advantages of such compounds are known, such as their biocompatibility, biodegradability, non-toxicity and excellent chemical resistance [36,37]. CS can easily be molded into various forms of substrates, such as films, porous scaffolds, nanofibers and hydrogels [38,39,40]. CS has previously been reported as a gel polymer electrolyte by some researchers, but it is mainly used in aqueous supercapacitors [41,42]. The hydrogen bonds of hydrophilic amino and hydroxyl groups in CS molecules causes poor dissolubility and poor affinity of the chitosan to liquid organic electrolytes, thus limiting the application of CS in alkali metal batteries [43,44,45]. In order to make chitosan as a GPE of lithium ion batteries a possibility, some researchers have attempted to blend chitosan with PEO or other materials [46]. Ai et al. blended CS with different mass fractions of PEO, in order to prepare a CS-LiTFSI-PEO composite polymer electrolyte with a three-dimensional cross-linked network structure [47]. Unfortunately, the electrolytes that are obtained by this method are hybrid solid polymer electrolytes based on CS substrates; as a result, they usually do not reach the 10^−3^ S cm^−1^ level of ionic conductivity [47,48]. Xu et al. hydrolyzed CS with hydrogen peroxide solution to obtain soluble small molecular chitosan, and then crosslinked it with polyethylene glycol diglycidyl (PEGGE) ether to obtain CS/PEG-composited GPE with a three-dimensional (3D) cross-linked network structure [49]. However, the molecular chain of CS breaks during hydrolysis to reduce the molecular weight, which also leads to a change in mechanical properties and subsequent electrochemical stability. Thus far, few investigations have been focused on modification of CS GPE on the basis of molecular structure.

In this study, a PEGylated chitosan was initially designed and prepared using a simple UV-induced reaction as a GPE polymer matrix for LIBs. PEGylated chitosan maintains good affinity for organic liquid electrolytes, making the chitosan-based organogel electrolyte possible and suitable for LIBs. Moreover, the complexation of the ether bond of PEG with lithium ions endows the chitosan matrix with the ability to rapidly transport lithium ions. Therefore, chitosan GPE films that are modified by grafting PEG molecules exhibit good properties, resulting in high ionic conductivity (>10^−3^ S·cm^−1^) and lithium ion transport numbers (>0.8) at ambient temperature.

## 2. Experimental

### 2.1. Materials

Poly (ethylene glycol) methyl ether methacrylate (PEGMA, Mn = 500 g mol^−1^) was purchased from Sigma-Aldrich, Shanghai, China. Chitosan [(C_6_H_11_NO_4_)_n_, deacetylation degree ≥ 95%], urea, chloropropene, lithium hydroxide, potassium hydroxide, sodium hydroxide, acetic acid, dimethyl sulfoxide (DMSO), dimethyl carbonate (DMC), ethylene carbonate (EC), N-methyl-2-pyrrolidone (NMP) and lithium bis (trifluoro-methane sulfonyl) imide (LiTFSI, 99%) were all purchased from Aladdin reagent company, Shanghai, China. Initiator 2959 was bought from BASF, Ludwigshafen, Germany. Poly-vinylidene fluoride (PVDF) was acquired from Solvay Pharmaceuticals, Shanghai, China, and Ketjen black carbon (KB) was obtained from Akzo Nobel, Shenzhen, China.

### 2.2. Preparation of Allyl-Modified Chitosan

The CS solvent was an aqueous solution that contained LiOH/KOH/urea/H_2_O at a mass ratio of 4.5:7:8:80.5. Under constant stirring, 9 g of CS powder was slowly added into 300 g of the above alkaline aqueous solution, and then placed at −40 °C overnight. Next, the frozen CS solution was thawed and stirred at room temperature until all the CS was dissolved to yield a clear CS solution. Afterwards, 23 mL of allyl chloride was slowly added to CS solution, and the solution was continuously stirred for 48 h in the dark. It was then neutralized with concentrated hydrochloric acid until that pH value reached 7, and then it was precipitated with acetone to obtain a milky flocculent allyl-modified chitosan. The resulting product was washed with deionized water, and freeze-dried to obtain the allyl-modified chitosan.

### 2.3. Preparation of PEGylated Chitosan (CS-g-PEG)

Firstly, 0.8 g of allyl-chitosan was dissolved in 40 mL of 2% acetic acid solution, and 0.009 g of initiator I2959 was dissolved in 200 μL of DMSO. The initiator I2959 and 1 mL of PEGMA were added to the allyl-chitosan solution, and after magnetic stirring for 10 min, the solution was placed into a UV reactor; UV radiation was used while stirring for 120 s, 240 s and 480 s, respectively. After the reaction was finished, the chitosan solution was neutralized using a sodium hydroxide solution and precipitating the modified chitosan, before washing and centrifuging the product using deionized water, and freeze-drying it to obtain the final product, CS-g-PEG. The products obtained after UV irradiation times of 120 s, 240 s and 480 s, were labeled as CS-g-PEG-120, CS-g-PEG-240, and CS-g-PEG-480, respectively.

### 2.4. Preparation of CS-g-PEG Gel Polymer Electrolytes

The CS-g-PEG film was obtained by dissolving 0.4 g of CS-g-PEG in 8 mL of DMSO solution, followed by casting in a mold and drying at 60 °C. The CS-g-PEG film was then air dried at room temperature. The obtained polymer film (CS-g-PEG) was punched into a disk (diameter of 14 mm) and immersed in an electrolyte solution (1 M LiTFSI DMC/EC = 1:1 volume) for 10 min to absorb solution and form the gel. The thickness of the dry film was 20–30 μm, and the thickness of the gel film was 70–80 μm.

### 2.5. Characterization of Allyl-Modified Chitosan and CS-g-PEG

The structures and chemical compositions of the allyl-modified chitosans, CS-g-PEG-120, CS-g-PEG-240 and CS-g-PEG-480, were tested using elemental analysis (Vario EL, Tokyo, Japan), nuclear magnetic resonance spectrometry, Shanghai, China. (^1^H-NMR, AVANCE NEO 400, 400 MHz with 2% acetic acid-d_4_ solution that was prepared by deuteroxide), Fourier transform infrared spectroscopy (FTIR, PerkinElmer Spectrum, Tokyo, Japan), and X-ray diffraction (XRD, Bruker AXS D2PHASER, Shanghai, China, 40 kV, 20 mA), at diffraction angles ranging from 10° to 80° with an increment of 0.01°.

The thermal stability was evaluated using thermogravimetric analysis (TGA, NETZSCH STA 449 F5/F3 Jupiter, Beijing, China.) under an N_2_ atmosphere, from 25 °C to 500 °C, at a heating rate of 10 °C min^−1^.

The electrolyte uptakes of AC and AC-PEGs were calculated according to Equation (1):

(1) $$n=\frac{(W_{t}-W_{0})\times 100\%}{W_{0}}$$

where $W_{0}$ and $W_{t}$ are the weights of the film before and after absorption of the liquid organic solvent, respectively.

### 2.6. Electrochemical Testing

The ionic conductivities of the CS-g-PEG GPEs were measured using electrochemical impedance spectroscopy (EIS, Biologic, VSP-300, Shanghai, China.), at frequencies from 10^6^ Hz to 10^−2^ Hz, an amplitude of 5 mV, and temperatures from 25 to 55 °C. The cells were assembled by sandwiching CS-g-PEG GPE films between two stainless steel (SS) plates. The ionic conductivity was calculated according to Equation (2):

(2) $$\sigma =\frac{L}{R_{b}\cdot S}$$

where *σ* is the ionic conductivity, *L* represents the thickness of the CS-g-PEG GPEs, and *R_b_* and *S* are the bulk ohmic resistance and area between CS-g-PEG GPE films and stainless steel, respectively.

The electrochemical windows of the CS-g-PEG GPEs were evaluated using cyclic voltammetry (CV) at 25 °C over a voltage range of −0.5 V to 5 V (vs. Li^+^/Li), at a scan rate of 1 mV s^−1^. The lithium ion transfer number ($t_{Li^{+}}$) was measured by combining EIS with chronoamperometry (CA) at room temperature, at an applied voltage of 10 mV. A symmetric cell was prepared by sandwiching CS-g-PEG GPEs films between two lithium metal electrodes. Calculation of $t_{Li^{+}}$ was carried out according to Equation (3):

(3) $$t_{Li^{+}}=\frac{I_{S}\left(U-R_{0}I_{0}\right)}{I_{0}\left(U-R_{S}I_{S}\right)}$$

where U is the applied voltage; $I_{0}$ and $I_{S}$ are the initial and steady state currents, respectively; $R_{0}$ and $R_{S}$ denote the initial and steady state Li/CS-g-PEG GPE interfacial resistances, respectively.

The dynamic interfacial stability between the GPEs and Li anodes at room temperature was evaluated via a galvanostatic cycling test of Li/CS-g-PEG/Li symmetric cells on a LAND CT 2001A battery test system, Shanghai, China. To this end, LiFePO_4_/GPEs/Li cells were assembled to measure the rate and long-term cycling performance over the charge–discharge voltage range of 2.5 V to 4.2 V. All batteries were prepared in an Ar-filled glove box at less than 0.1 ppm H_2_O and O_2_. LiFePO_4_ powders with KB carbon and PVDF binder solution in a mass ratio of 80:10:10 (wt %) were prepared and coated on Al foils. LiFePO_4_ cathodes were punched into disks (diameters of 10 mm) and dried in a vacuum at 110 °C for 12 h. The lithium iron phosphate loading was approximately 2.3–2.8 mg cm^−2^ per cathode.

## 3. Results and Discussion

**Scheme 1:**
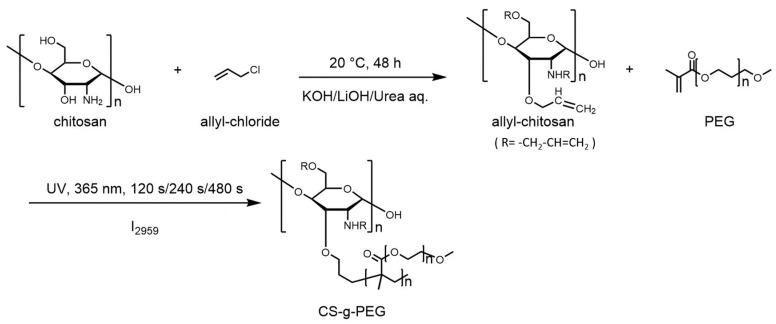
Reaction mechanism of allyl-modified chitosan and CS-g-PEG.

**Figure 1:**
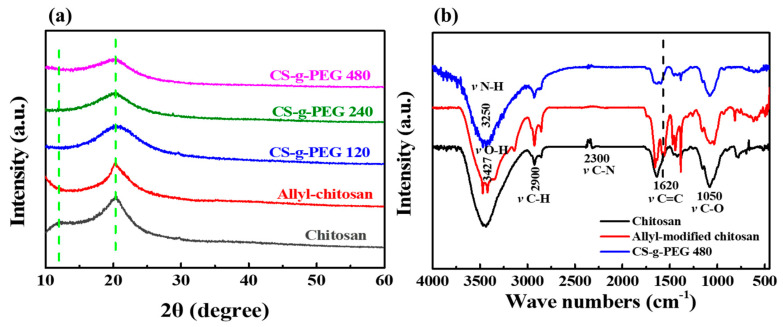
(**a**) X-ray diffraction (XRD) patterns of chitosan, allyl-chitosan and CS-g-PEG 120, 240 and 480, and (**b**) Fourier-transform infrared (FTIR) spectra of chitosan, allyl-chitosan and CS-g-PEG.

**Figure 2:**
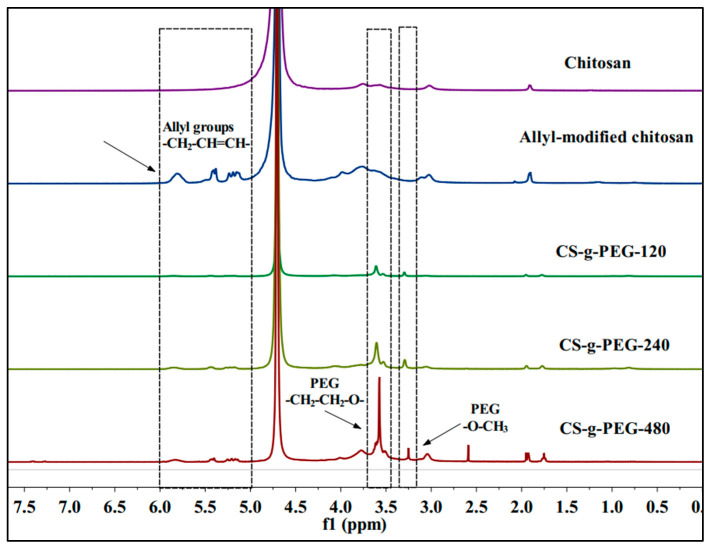
^1^H-NMR spectra of chitosan, allyl-chitosan and CS-g-PEG 120, 240 and 480 in 2% acetic acid-d_4_ solution that was prepared using deuteroxide at 400 MHz.

As shown in Scheme 1, the PEGylation of chitosan was achieved using a simple two-step reaction. The allyl functional group was initially attached to the chitosan backbone via a substitution reaction in an alkaline solution to obtain allyl-modified chitosan. Next, the PEGMA soft chain was grafted onto allyl chitosan using free radical polymerization under UV radiation and photoinitiator I2959 to obtain PEGylated chitosan. The chemical compositions and structures of chitosan, allyl-modified chitosan and CS-g-PEGs were characterized by XRD, FTIR and ^1^H-NMR. Firstly, the chitosan before and after modification was characterized via XRD to test the change in crystallinity. As shown in Figure 1a, the XRD pattern of the chitosan powder before modification shows sharp diffraction peaks at about 11.8° and 20.3°, indicating the existence of a crystalline region in chitosan. This was also verified and reported in many other studies [50,51]. After allyl modification, the crystallization peak at 20.3° decreased rapidly and the peak at 11.8° disappeared completely, which means that the crystallinity of allyl-modified CS decreased, and the intermolecular forces were weakened by chemical modification. Meanwhile, the introduction of PEG further resulted in a decrease in the crystallinity in CS. The reduced crystallinity thereby improved the flexibility of the molecular chain, which was favorable for transporting lithium ions. As displayed in Figure 1b, the IR spectrum of allyl-modified chitosan showed a characteristic peak at 1625 cm^−1^ which corresponded to a stretching mode of the C=C bond of allyl groups. Further absorption peaks also appeared at 1108 cm^−1^ and 2895 cm^−1^, and were assigned to the C–O–C and C–H vibrations of chitosan, respectively. The spectrum of CS-g-PEG showed a new characteristic peak at 1750 cm^−1^ that was attributed to the C=O vibration obtained from PEGMA after the grafting reaction. Meanwhile, the characteristic peak of CS-g-PEG is located at 1625 cm^−1^, which is connected with the vibration mode of C=C bond; however, its intensity is weaker than that of allyl modified chitosan. This indicates that the C=C bond in allyl-modified CS was gradually consumed after reaction with PEGMA with different reaction times. Thus, FTIR analysis confirmed the successful PEGylation of chitosan. These results were also confirmed by ^1^H-NMR spectra. As shown in Figure 2, the peak at 3.52 ppm was assigned to the proton peak of –CH_2_–CH_2_–O–, and the peak at 3.25 ppm was assigned to the proton peak of –O–CH_3_ within the PEGMA chains. These new peaks were both detected after grafting the PEGMA fragment onto chitosan. In addition, the proton peak intensity increased with increasing UV irradiation time.

**Figure 3:**
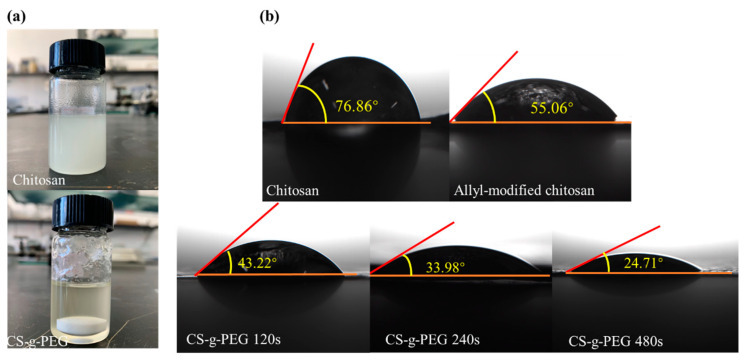
(**a**) Photographs of chitosan and CS-g-PEG dissolved in DMSO. (**b**) Contact angle measurements between liquid electrolyte and chitosan, allyl-chitosan and CS-g-PEG 120, 240 and 480 films.

In addition, it was confirmed through elemental analysis (Table 1) that the grafting degree of PEG can be controlled by UV irradiation time. The degree of grafting herein was defined by the molar ratio of grafted PEG to chitosan monomer. In our experiment, the grafting ratio of allyl was 35.17%, and the grafting degree of PEG increased from 7.26% to 20.46% when the reaction time increased from 120 s to 480 s. As shown in Figure 3a, the chitosan powder and the PEGylated CS-g-PEG sample were dissolved in DMSO solution, respectively, to test their affinity to organic solvents. It can be seen that the unmodified chitosan was completely insoluble in DMSO, and showed a phase separation state, while the PEGylated CS was easy to dissolve in DMSO solution as a nearly clear solution. This indicated that CS-g-PEG possesses superior affinity to organic solvent, which is beneficial to converting it into a gel polymer electrolyte for LIBs. In addition, the wettability of chitosan, allyl-modified chitosan and CS-g-PEG films to liquid electrolyte was evaluated via contact angle measurements. The results are shown in Figure 3b. The contact angle between the pure chitosan film and the liquid electrolyte was 76.86°, indicating that the affinity of the pure chitosan film to the liquid electrolyte was very poor. The contact angle of allyl-modified chitosan film was 55.06°, which was lower than that of chitosan film; this indicated that the affinity of the allyl-modified chitosan film to the liquid electrolyte is better than that of chitosan film. Fortunately, the contact angles of CS–g–PEG–120, 240 and 480 films were 43.22°, 33.98° and 24.71°, respectively, which indicated that the affinity of CS-g-PEG films to liquid electrolyte became better with an increase in UV irradiation time.

**Figure 4:**
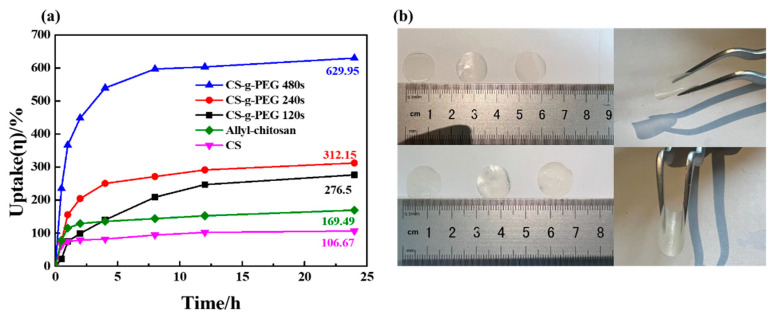
(**a**) Swelling properties of chitosan, allyl-chitosan and CS-g-PEG 120, 240 and 480 films after 24 h immersion in electrolytes. (**b**) Photographs of the films before and after swelling in liquid electrolyte.

As shown in Figure 4a, all of the films were immersed in the liquid electrolyte for 24 h, and then the weight change of the films was measured using a microbalance to test the swelling property of the films at room temperature. It was found that the swelling equilibrium of chitosan, allyl-chitosan and CS-g-PEG films could be reached within 24 h; however, the equilibrium swelling ratios were quite different. Among them, the chitosan film had the lowest electrolyte absorption, corresponding to a weight gain of 106.67%; since a large number of polar functional groups and hydrogen bonds exist in the chitosan molecule, this resulted in poor compatibility with carbonate ester electrolyte solutions. The electrolyte absorption of allyl-chitosan film was 169.49%, which is slightly higher than that of chitosan film, because a part of hydrogen bonds in the molecule were broken as a result of the grafting of C=C bonds. In contrast, the electrolyte absorption of CS-g-PEG-480 was 629.95%, with increasing grafting degree, indicating that the grafting degree of PEG had a significant effect on the affinity of the film to liquid electrolyte. As shown in Figure 4b, we took images of the films before and after swelling in liquid silicate electrolyte for 24 h; after swelling equilibrium which reached, the integrity of the film remained good, and the size became slightly larger. In addition, as shown in Figure 4, the film had good flexibility and bendability after swelling.

**Figure 5:**
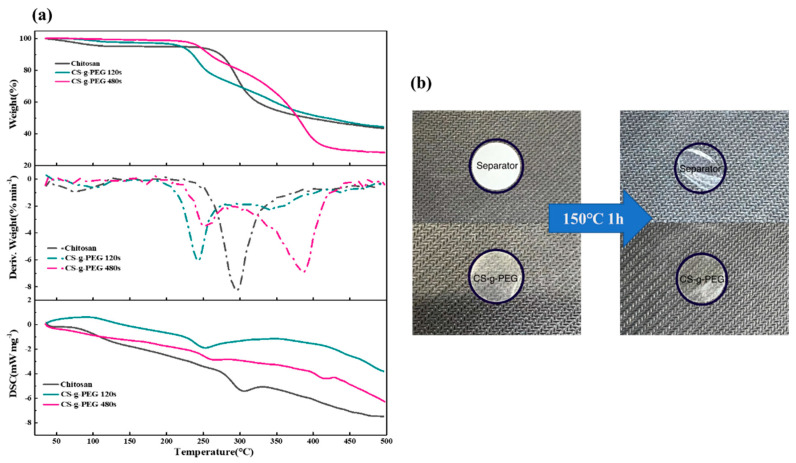
(**a**) Weight losses of films, the differential thermogravimetry (TG) curves and the differential scanning calorimetry (DSC) curves at temperatures from 25 to 600 °C. (**b**) Images of commercial separator Celgard 2500 and CS-g-PEG that were kept at 150 °C for 2 h.

As an important parameter of gel polymer electrolytes, thermal stability has great influence on the safety of lithium batteries. Figure 5a shows the weight losses of films, the differential thermogravimetry (TG) curves and the differential scanning calorimetry (DSC) curves at temperatures from 25 to 600 °C. Chitosan had a weight loss phase in the temperature range of 25–500 °C, at about 300 °C, corresponding to the breakdown of chitosan molecules. However, a new weight loss phase appeared in CS-g-PEG at around 400 °C, which corresponds to the decomposition of the PEG grafted onto the main chain of chitosan, and the weight loss increased with an increase in grafting. In CS-g-PEG, the weight loss phase resulting from the decomposition of chitosan reduced from around 300 °C to around 240 °C, probably due to the disruption of hydrogen bonds in the chitosan molecule caused by grafting. In addition, we measured differential scanning calorimetry (DSC) curves of chitosan and CS-g-PEG over the temperature range of 25–500 °C to further reveal the thermodynamic changes that occurred during the heating experiments. The two main heat absorption peaks in the graph occur at around 300 °C and 400 °C, caused by the breakdown of the chitosan and PEG chains, respectively, which are consistent with the TG curves. Although the decomposition temperature of chitosan decreased to some extent with the grafting of PEG, it still had a significant advantage in terms of thermal stability compared to current commercial battery separators, which have a dissolution temperature of approximately 165 °C [52,53]. A more intuitive heating experiment was also carried out, in which the commercial Celgard 2500 separator, Shanghai, China and the CS-g-PEG films were each heat-treated at 150 °C for 1 h. In Figure 5b, we can see that after 1 h at 150 °C, the CS-g-PEG film remained intact in size and shape, with no significant changes. In contrast, the commercial Celgard 2500 separator showed shrinkage, and a change in color from opaque to transparent. All of the above thermal data show that the CS-g-PEG film has good thermal stability, and its excellent thermal stability contributes to the safety of lithium ion batteries.

**Figure 6:**
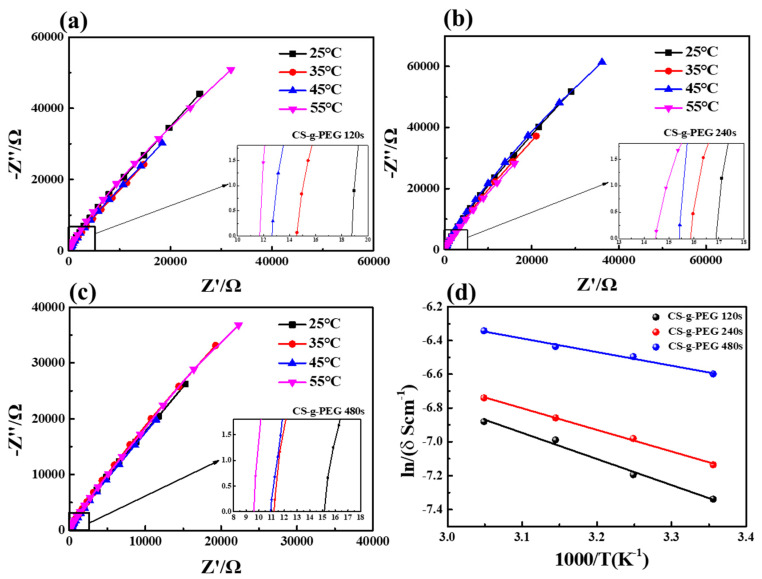
Impedance plots of ACMC GPE membranes at different temperatures: (**a**) CS-g-PEG-120, (**b**) CS-g-PEG-240, (**c**) CS-g-PEG-480 and (**d**) Arrhenius plots of CS-g-PEG films.

When polymers are used as GPE for LIBs, their ionic conductivity at room temperature is critical to the performance of LIBs [54]. Here, the temperature-dependent ionic conductivities of CS-g-PEG films with different illumination times were measured using AC impedance spectroscopy in the range of 25 °C to 55 °C, in order to evaluate the effect of PEG grafting degree on the electrochemical performance of assembled lithium ion batteries. The results are shown in Figure 6. It is worth noting that we did not provide electrochemical data for chitosan and allyl-modified chitosan films here because their poor affinity to liquid electrolyte makes them difficult to form a gel state, which prevents the ionic conductivity tests from being carried out. It can be seen from Figure 6 and Table 2 that the ionic conductivity of the CS-g-PEG films at 25 °C increases from 0.65 × 10^−3^·S·cm^−1^ to 1.12 × 10^−3^ S cm^−1^ with an increase in the amount of PEG. Moreover, as the temperature increased from 25 °C to 55 °C, the ionic conductivity of all CS-g-PEG films increased, showing typical Arrhenius behavior. The activation energy of the CS-g-PEG films with different grafting degrees can be calculated and fitted using the Arrhenius formula, $\sigma =\sigma_{0}\exp\left(-\frac{E_{a}}{kT}\right)$, where $E_{a}$ is the activation energy, $\sigma_{0}$ is the pre-exponential factor, *k* denotes the Boltzmann constant, and *T* is the experiment temperature. The activation energies for CS-g-PEG-120-, CS-g-PEG-240- and CS-g-PEG-480-based GPEs obtained by calculation and fitting were 12.33, 10.63, and 9.85 kJ·mol^−1^, respectively. This suggests that the introduction of PEG segments probably plays a key role in enhancing lithium ion conduction, and the ether bonds of PEG can coordinate and dissociate with lithium ions to facilitate the transport of lithium ions in the gel films. The higher grafting degree of CS-g-PEG-480 has more PEG segments, which makes transport of lithium ions in the gel films more efficient.

**Figure 7:**
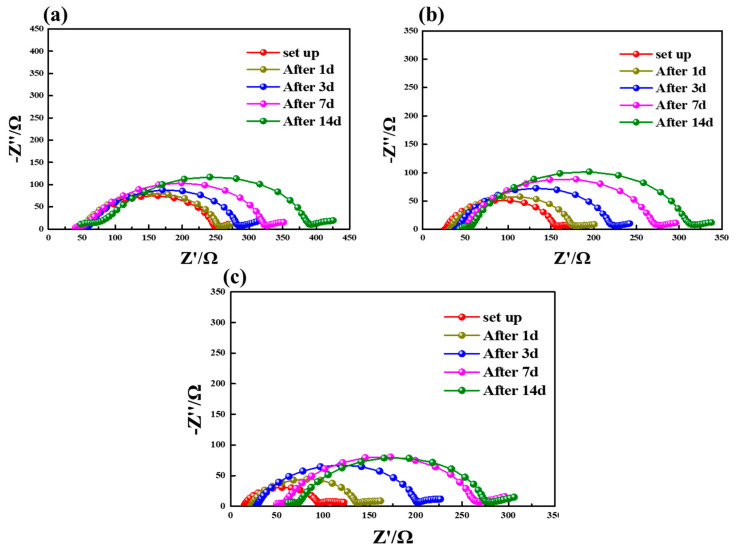
Nyquist plots of Li/GPE/Li cells with GPEs of (**a**) CS-g-PEG-120, (**b**) CS-g-PEG-240, (**c**) CS-g-PEG-480, on days 1, 3, 5, 7 and 14.

The interfacial resistance (*R_i_*) between the gel polymer electrolyte and the electrode has a significant effect on the performance of the lithium ion battery [55]. Li/GPEs/Li symmetric cells were assembled at 25 °C to test the interfacial compatibility of CS-g-PEG film with electrodes, and its change in 14 days. As shown in Figure 7, that cell impedance plot shows two semicircles at high and medium frequency, which correspond to the passivation layer resistance (*R_p_*) and the charge transfer resistance (*R_ct_*), respectively. The high frequency intercept of the real axis corresponds to the bulk resistance (*R_0_*). It can be seen from the figures that all of the CS-g-PEG films with different grafting times have very low interfacial resistance, and the interfacial resistance of CS-g-PEG-480 is about 100 Ω, which is the lowest recorded value among the products. It was found that the interfacial resistance decreased with an increase in the PEG content. It can be concluded that the higher the amount of PEG grafted, the better the absorbency of the gel film for liquid electrolyte, which leads to better interfacial compatibility between the film and the electrode. At the same time, the assembled cells were placed at room temperature for 14 days, and the change of interfacial resistance was measured. It was found that the interfacial resistance increased gradually with an increase in time, and the cell assembled by CS-g-PEG-480 reached a relatively stable state in about 7 days. This demonstrated the stable interface that formed between GPE film and lithium electrodes.

**Figure 8:**
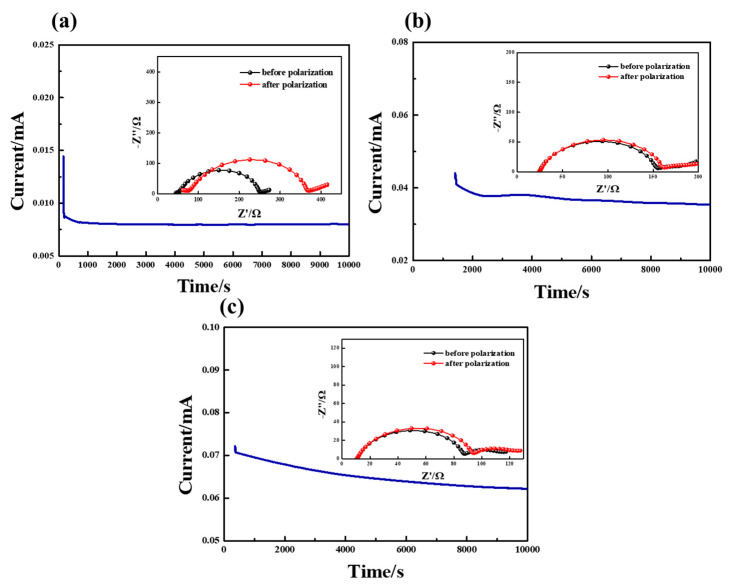
Current–time curves of DC polarization and Nyquist plots before and after polarization in different GPEs. (**a**) CS-g-PEG-120, (**b**) CS-g-PEG-240, (**c**) CS-g-PEG-480.

**Scheme 2:**
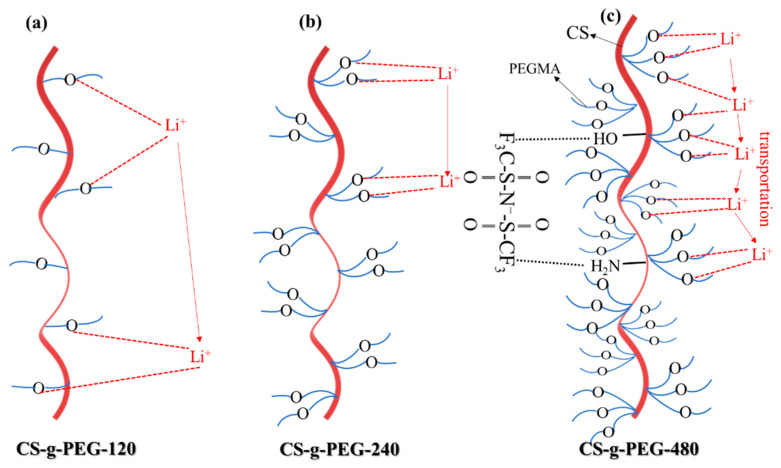
The transport process of lithium ions in CS-g-PEG molecules.

For GPE, the lithium ion transport number ($t_{Li^{+}}$) is very important to represent the transport properties of lithium ions in the electrolyte. High $t_{Li^{+}}$ values can effectively lead to a reduction in the polarization effect and an inhibition of lithium dendrite growth during charge/discharge cycles of a lithium ion battery; it can result in improved energy output of the cell. Figure 8 and Table 2 present the Li^+^ transport numbers based on CS-g-PEG films that were obtained by combining the EIS technique with the chronoamperometry (CA) technique at room temperature. The optimum Li^+^ transport number of CS-g-PEG-480 at room temperature is 0.816, which is much higher than that of liquid electrolyte. The Li^+^ transfer number increased from 0.58 to 0.816 with an increase in PEG grafting degree, which was consistent with the change in ionic conductivity; this indicates that the introduction of PEG segments was beneficial to the transport property of lithium ions in the battery. The excellent Li^+^ transfer ability of CS-g-PEG is firstly due to the introduction of PEG chain segments. Secondly, the introduction of PEG chains breaks the hydrogen bonds originally formed in chitosan molecules, resulting in residual amino and hydroxyl groups on the chitosan main chain that can interact with the anions of lithium salt to fix the movement of some anions (Scheme 2). In addition, as shown in Scheme 2, more PEG chain segments are available on CS-g-PEG-480 with a higher grafting degree, so that more Li^+^ can be transported in the GPE films, and more Li^+^ to Li^+^ transport bridges can be constructed [33].

**Figure 9:**
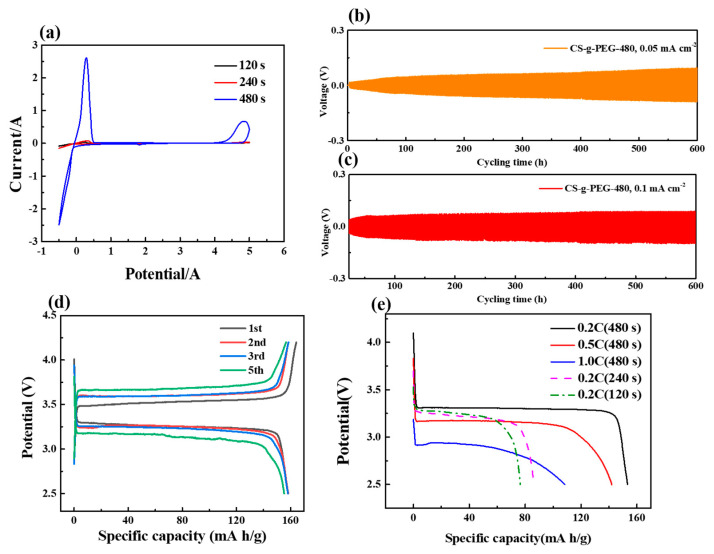
Electrochemical performance of CS-g-PEG GPEs. (**a**) CV curves of stainless steel (SS)/CS-g-PEG GPEs/Li cells. (**b**) Voltage profiles of Li/CS-g-PEG-480/Li cells at transfer charges of 0.2 mA·h cm^−2^, 0.05 mA cm^−2^ and (**c**) 0.1 mA cm^−2^ current densities. (**d**) Charge–discharge profiles of LiFePO_4_/Li battery using CS-g-PEG-480 at 0.2 C for different cycles. (**e**) The initial discharge profiles of LiFePO_4_/Li battery using CS-g-PEG-120, 240 and 480.

Figure 9a Evaluation of the electrochemical stability of CS-g-PEG-based GPE using linear sweep voltammograms (LSV) of Li/GPE/SS cells. The electrolyte with CS-g-PEG-120 and CS-g-PEG-240 decomposes at about 4.9 V, while the electrolyte with CS-g-PEG-480 decomposes at about 4.75 V and has a higher oxidation peak, which may be a result of the higher reactivity of CS-g-PEG-480 due to the more PEG segments on it [25]. The result indicates that CS-g-PEG can maintain excellent anodic stability at high voltages, and is a very promising GPE material for application.

The interfacial compatibility of CS-g-PEG with lithium anode in symmetric Li/GPE/Li batteries at different current densities and cycles was also measured via the galvanostatic charge/discharge method. As shown in Figure 9b, the Li/CS-g-PEG-480/Li cell exhibited an initial overpotential of 0.01 V at a current density of 0.05 mA cm^−2^, followed by a slow polarization; finally, the overpotential stabilized below 0.1 V within 600 h. At a current density of 0.1 mA cm^−2^, the overpotential of the Li/CS-g-PEG-480/Li cell remained at around 0.05 V for 600 h, and showed a more stable state, as shown in Figure 9c. These data indicate that CS-g-PEG-480 has good cycling stability with a lithium anode.

Finally, a coin cell Li/GPE/LiFePO_4_ was assembled to test the cycling performance of the cell. Figure 9d shows the charge–discharge curve for the CS-g-PEG-480-based coin cell at 0.2 C. The initial discharge capacity of the Li/CS-g-PEG-480/LiFePO_4_ battery was 158 mA h g^−1^, and it still had a discharge capacity of 155 mA h g^−1^ after five cycles. Figure 9e shows the discharge curves of Li/CS-g-PEG/LiFePO_4_ cells with different PEG grafting degrees. The data selected are the initial discharge capacity of each cell at 0.2 C/0.5 C/1 C. It can be seen that the initial discharge capacity of the CS-g-PEG-480-based cell was 142 mA h g^−1^ at 0.5 C and 108 mA h g^−1^ at 1 C, which may be due to the more severe polarization occurring inside the electrolyte at higher current densities. In addition, the initial discharge capacity of the CS-g-PEG-240-based battery was 85 mA h g^−1^, and the initial discharge capacity of the CS-g-PEG-120-based battery was 78 mA h g^−1^. The lower specific discharge capacity of the Li/CS-g-PEG/LiFePO_4_ battery with lower grafting degree may be due to its relatively low lithium ion transfer number and ionic conductivity. However, in general, CS-g-PEG-480 still exhibits great application potential for GPE-based LIBs.

## 4. Conclusions

Using cheap and sustainable chitosan as a raw material, a PEGylated chitosan gel electrolyte was successfully designed and synthesized using a simple and low-cost grafting reaction. The UV irradiation time was used as a control variable to obtain PEGylated chitosan gel electrolytes with different PEG grafting degrees. The affinity of PEGylated chitosan to liquid electrolyte significantly improved, and the application requirement of the lithium ion battery was met. The complexation of the ether bond of PEG with lithium ions provided the chitosan matrix with the ability to transport lithium ions rapidly; thus, the button cell that was assembled with CS-g-PEG gel film achieved an ionic conductivity of 1.12 × 10^−3^ S cm^−1^, and a lithium ion transport number of 0.816, at room temperature. In addition, the initial discharge capacity of the coin cell that was assembled with CS-g-PEG-480 at room temperature was able to reach 158 mA h g^−1^; its long cycle performance is worth further exploration. In summary, the designed chitosan-based GPE has great prospects in the field of energy storage, and is expected to further replace the use of liquid electrolytes and solve the safety problem that is associated with them.

## Figures and Tables

**Table 1:** Elemental analysis of allyl-chitosan and CS-g-PEG 120, 240 and 480.

Name	Weight(mg)	N%	C%	H%	Grafting Rate
Allyl-chitosan	1.97	6.87	37.89	6.125	35.17%
CS-g-PEG (120 s)	2.33	6.63	41.57	6.674	7.26%
CS-g-PEG (240 s)	1.829	6.2	42.72	6.64	10.26%
CS-g-PEG (480 s)	2.083	5.06	42.45	6.738	20.46%

**Table 2:** Lithium ion transport numbers and ionic conductivities of CS-g-PEG.

Name	$t_{Li^{+}}$	δ × 10^−3^(S cm^−1^)/25 °C	δ × 10^−3^(S cm^−1^)/35 °C	δ × 10^−3^(S cm^−1^)/45 °C	δ × 10^−3^(S cm^−1^)/55 °C
CS-g-PEG (120 s)	0.58	0.65	0.83	0.95	1.03
CS-g-PEG (240 s)	0. 60	0.88	0.93	0.97	1.07
CS-g-PEG (480 s)	0.816	1.12	1.51	1.55	1.76

## Data Availability

The data presented in this study are available on request from the corresponding author.

## References

[1] Li M., Lu J., Chen Z., Amine K. (2018). 30 Years of Lithium-Ion Batteries. Adv. Mater..

[2] Lin D., Liu Y., Cui D.L.Y.L.Y. (2017). Reviving the lithium metal anode for high-energy batteries. Nat. Nanotechnol..

[3] Wu F., Maier J., Yu Y. (2020). Guidelines and trends for next-generation rechargeable lithium and lithium-ion batteries. Chem. Soc. Rev..

[4] Xu W., Chen X., Ding F., Xiao J., Wang D., Pan A., Zheng J., Li X.S., Padmaperuma A.B., Zhang J.-G. (2012). Reinvestigation on the state-of-the-art nonaqueous carbonate electrolytes for 5 V Li-ion battery applications. J. Power Sources.

[5] Yue H., Yang Y., Xiao Y., Dong Z., Cheng S., Yin Y., Ling C., Yang W., Yu Y., Yang S. (2019). Boron additive passivated carbonate electrolytes for stable cycling of 5 V lithium–metal batteries. J. Mater. Chem. A.

[6] Yang H., Li J., Sun Z., Fang R., Wang D.-W., He K., Cheng H.-M., Li F. (2020). Reliable liquid electrolytes for lithium metal batteries. Energy Storage Mater..

[7] Fan W., Li N.-W., Zhang X., Zhao S., Cao R., Yin Y., Xing Y., Wang J., Guo Y.-G., Li C. (2018). A Dual-Salt Gel Polymer Electrolyte with 3D Cross-Linked Polymer Network for Dendrite-Free Lithium Metal Batteries. Adv. Sci..

[8] Li X., Zheng Y., Pan Q., Li C.Y. (2019). Polymerized Ionic Liquid-Containing Interpenetrating Network Solid Polymer Electrolytes for All-Solid-State Lithium Metal Batteries. ACS Appl. Mater. Interfaces.

[9] Lian P.-J., Zhao B.-S., Zhang L.-Q., Xu N., Wu M.-T., Gao X.-P. (2019). Inorganic sulfide solid electrolytes for all-solid-state lithium secondary batteries. J. Mater. Chem. A.

[10] Dirican M., Yan C., Zhu P., Zhang X. (2019). Composite solid electrolytes for all-solid-state lithium batteries. Mater. Sci. Eng. R Rep..

[11] Ke X., Wang Y., Ren G., Yuan C. (2020). Towards rational mechanical design of inorganic solid electrolytes for all-solid-state lithium ion batteries. Energy Storage Mater..

[12] Cheng X., Pan J., Zhao Y., Liao M., Peng H. (2018). Gel Polymer Electrolytes for Electrochemical Energy Storage. Adv. Energy Mater..

[13] Long M.-C., Wang T., Duan P.-H., Gao Y., Wang X.-L., Wu G., Wang Y.-Z. (2022). Thermotolerant and fireproof gel polymer electrolyte toward high-performance and safe lithium-ion battery. J. Energy Chem..

[14] Jankowsky S., Hiller M., Stolina R., Wiemhöfer H.-D. (2015). Performance of polyphosphazene based gel polymer electrolytes in combination with lithium metal anodes. J. Power Sources.

[15] Jin M., Zhang Y., Yan C., Fu Y., Guo Y., Ma X. (2018). High-Performance Ionic Liquid-Based Gel Polymer Electrolyte Incorporating Anion-Trapping Boron Sites for All-Solid-State Supercapacitor Application. ACS Appl. Mater. Interfaces.

[16] Kuo P.-L., Wu C.-A., Lu C.-Y., Tsao C.-H., Hsu C.-H., Hou S.-S. (2014). High Performance of Transferring Lithium Ion for Polyacrylonitrile-Interpenetrating Crosslinked Polyoxyethylene Network as Gel Polymer Electrolyte. ACS Appl. Mater. Interfaces.

[17] Zheng Z., Gao X., Luo Y., Zhu S. (2016). Employing Gradient Copolymer To Achieve Gel Polymer Electrolytes with High Ionic Conductivity. Macromolecules.

[18] Li W., Pang Y., Liu J., Liu G., Wang Y., Xia Y. (2017). A PEO-based gel polymer electrolyte for lithium ion batteries. RSC Adv..

[19] Kim Y., Kwon S.J., Jang H.-K., Jung B.M., Lee S.B., Choi U.H. (2017). High Ion Conducting Nanohybrid Solid Polymer Electrolytes via Single-Ion Conducting Mesoporous Organosilica in Poly(ethylene oxide). Chem. Mater..

[20] Yan X., Peng B., Hu B., Chen Q. (2016). PEO-urea-LiTFSI ternary complex as solid polymer electrolytes. Polymer.

[21] Tsao C.-H., Kuo P.-L. (2015). Poly(dimethylsiloxane) hybrid gel polymer electrolytes of a porous structure for lithium ion battery. J. Membr. Sci..

[22] Wang S.-H., Kuo P.-L., Hsieh C.-T., Teng H. (2014). Design of Poly(Acrylonitrile)-Based Gel Electrolytes for High-Performance Lithium Ion Batteries. ACS Appl. Mater. Interfaces.

[23] Hosseinioun A., Nürnberg P., Schönhoff M., Diddens D., Paillard E. (2019). Improved lithium ion dynamics in crosslinked PMMA gel polymer electrolyte. RSC Adv..

[24] Yang D., He L., Liu Y., Yan W., Liang S., Zhu Y., Fu L., Chen Y., Wu Y. (2019). An acetylene black modified gel polymer electrolyte for high-performance lithium–sulfur batteries. J. Mater. Chem. A.

[25] Fu F., Zheng Y., Jiang N., Liu Y., Sun C., Zhang A., Teng H., Sun L., Xie H. (2022). A Dual-Salt PEO-based polymer electrolyte with Cross-Linked polymer network for High-Voltage lithium metal batteries. Chem. Eng. J..

[26] Lu Q., Dong L., Chen L., Fu J., Shi L., Li M., Zeng X., Lei H., Zheng F. (2020). Inorganic-organic gel electrolytes with 3D cross-linking star-shaped structured networks for lithium ion batteries. Chem. Eng. J..

[27] Safa M., Chamaani A., Chawla N., El-Zahab B. (2016). Polymeric Ionic Liquid Gel Electrolyte for Room Temperature Lithium Battery Applications. Electrochim. Acta.

[28] Qiu F., Huang Y., Hu X., Li B., Zhang X., Luo C., Li X., Wang M., Wu Y., Cao H. (2019). An Ecofriendly Gel Polymer Electrolyte Based on Natural Lignocellulose with Ultrahigh Electrolyte Uptake and Excellent Ionic Conductivity for Alkaline Supercapacitors. ACS Appl. Energy Mater..

[29] Huang B.Y., Zhang Y.D., Que M.M., Xiao Y.B., Jiang Y.Q., Yuan K., Chen Y.W. (2017). A facile in situ approach to ion gel based polymer electrolytes for flexible lithium batteries. RSC Adv..

[30] Hu X., Fan L., Qin G., Shen Z., Chen J., Wang M., Yang J., Chen Q. (2019). Flexible and low temperature resistant double network alkaline gel polymer electrolyte with dual-role KOH for supercapacitor. J. Power Sources.

[31] Na R., Wang X., Lu N., Huo G., Lin H., Wang G. (2018). Novel egg white gel polymer electrolyte and a green solid-state supercapacitor derived from the egg and rice waste. Electrochim. Acta.

[32] Yu F., Zhang H., Zhao L., Sun Z., Li Y., Mo Y., Chen Y. (2020). A flexible Cellulose/Methylcellulose gel polymer electrolyte endowing superior Li^+^ conducting property for lithium ion battery. Carbohydr. Polym..

[33] Zhang H., Wang S., Wang A., Li Y., Yu F., Chen Y. (2022). Polyethylene glycol-grafted cellulose-based gel polymer electrolyte for long-life Li-ion batteries. Appl. Surf. Sci..

[34] Selvanathan V., Ruslan M.H., Alkahtani A.A.N., Amin N., Sopian K., Muhammad G. (2021). Akhtaruzzaman Organosoluble, esterified starch as quasi-solid biopolymer electrolyte in dye-sensitized solar cell. J. Mater. Res. Technol..

[35] Divya K., Jisha M.S. (2017). Chitosan nanoparticles preparation and applications. Environ. Chem. Lett..

[36] Jin T., Liu T., Lam E., Moores A. (2021). Chitin and chitosan on the nanoscale. Nanoscale Horiz..

[37] Benchamas G., Huang G., Huang S., Huang H. (2021). Preparation and biological activities of chitosan oligosaccharides. Trends Food Sci. Technol..

[38] Zhang Q., Chen Y., Wei P., Zhong Y., Chen C., Cai J. (2021). Extremely strong and tough chitosan films mediated by unique hydrated chitosan crystal structures. Mater. Today.

[39] Takeshita S., Zhao S., Malfait W.J. (2021). Transparent, Aldehyde-Free Chitosan Aerogel. Carbohydr. Polym..

[40] Crini G. (2019). Historical review on chitin and chitosan biopolymers. Environ. Chem. Lett..

[41] Zhang Q., Zhao L., Yang H., Kong L., Ran F. (2021). Alkali-tolerant polymeric gel electrolyte membrane based on cross-linked carboxylated chitosan for supercapacitors. J. Membr. Sci..

[42] Li C., Liu G., Wang S., Wang D., Liu F., Cui Y., Liang D., Wang X., Yong Z., Chi Y. (2022). Polyvinyl alcohol/quaternary ammonium chitosan hydrogel electrolyte for sensing supercapacitors with excellent performance. J. Energy Storage.

[43] Cheng Q., Zhang Y., Zheng X., Sun W., Li B., Wang D., Li Z. (2021). High specific surface crown ether modified chitosan nanofiber membrane by low-temperature phase separation for efficient selective adsorption of lithium. Sep. Purif. Technol..

[44] Kim S., Cho M., Lee Y. (2020). Multifunctional Chitosan–rGO Network Binder for Enhancing the Cycle Stability of Li–S Batteries. Adv. Funct. Mater..

[45] Nowacki K., Galiński M., Stępniak I. (2019). Synthesis and characterization of modified chitosan membranes for applications in electrochemical capacitor. Electrochim. Acta.

[46] Li C., Huang Y., Chen C., Feng X., Zhang Z., Liu P. (2022). A high-performance solid electrolyte assisted with hybrid biomaterials for lithium metal batteries. J. Colloid Interface Sci..

[47] Ai S., Wang T., Li T., Wan Y., Xu X., Lu H., Qu T., Luo S., Jiang J., Yu X. (2020). A Chitosan/Poly(ethylene oxide)-Based Hybrid Polymer Composite Electrolyte Suitable for Solid-State Lithium Metal Batteries. ChemistrySelect.

[48] Ai S., Mazumdar S., Li H., Cao Y., Li T. (2021). Nano-silica doped Composite Polymer Chitosan/Poly(ethylene oxide)-Based Electrolyte with High Electrochemical Stability Suitable for Quasi Solid-state Lithium Metal Batteries. J. Electroanal. Chem..

[49] Xu D., Jin J., Chen C., Wen Z. (2018). From Nature to Energy Storage: A Novel Sustainable 3D Cross-Linked Chitosan–PEGGE-Based Gel Polymer Electrolyte with Excellent Lithium-Ion Transport Properties for Lithium Batteries. ACS Appl. Mater. Interfaces.

[50] Cui L., Gao S., Song X., Huang L., Dong H., Liu J., Chen F., Yu S. (2018). Preparation and characterization of chitosan membranes. RSC Adv..

[51] Chae K.-S., Shin C.-S., Shin W.-S. (2018). Characteristics of cricket (*Gryllus bimaculatus*) chitosan and chitosan-based nanoparticles. Food Sci. Biotechnol..

[52] Yu L., Gu J., Pan C., Zhang J., Wei Z., Zhao Y. (2022). Recent developments of composite separators based on high-performance fibers for lithium batteries. Compos. Part A Appl. Sci. Manuf..

[53] Zhang J., Liu Z., Kong Q., Zhang C., Pang S., Yue L., Wang X., Yao J., Cui G. (2013). Renewable and Superior Thermal-Resistant Cellulose-Based Composite Nonwoven as Lithium-Ion Battery Separator. ACS Appl. Mater. Interfaces.

[54] Benouar A., Bacha M.R.A. (2020). Ionic Conductivity of Chitosan-Lithium Electrolyte in Biodegradable Battery Cell. Indones. J. Chem..

[55] Single F., Horstmann B., Latz A. (2019). Theory of Impedance Spectroscopy for Lithium Batteries. J. Phys. Chem. C.

